# Cardiac Function and Diastolic Dysfunction in Behcet's Disease: A Systematic Review and Meta-Analysis

**DOI:** 10.1155/2016/9837184

**Published:** 2016-05-10

**Authors:** Fawad Aslam, Salman J. Bandeali, Cynthia Crowson, Mahboob Alam

**Affiliations:** ^1^Division of Rheumatology, Mayo Clinic Health System, Eau Claire, WI 54703, USA; ^2^Department of Cardiology, Texas Heart Institute at CHI St. Luke's Medical Center, Houston, TX 77030, USA; ^3^Division of Biomedical Statistics and Informatics, Department of Health Sciences Research and Division of Rheumatology, Department of Internal Medicine, Mayo Clinic, Rochester, MN 55906, USA; ^4^Section of Cardiology, Department of Medicine, Baylor College of Medicine, Houston, TX 77030, USA

## Abstract

*Background*. Cardiovascular involvement in Behcet's disease (BD) is reported and has variable manifestations. It is not clear if diastolic dysfunction (DD) is increased in BD. Our objective was to evaluate the existing literature to determine if cardiac dysfunction, particularly DD, was more prevalent in these patients.* Methods*. A systematic review and meta-analysis of the available studies analyzing the echocardiographic findings in BD was conducted using a random-effects model. Mean differences were used to calculate the effect sizes of the echocardiographic parameters of interest.* Results*. A total of 22 studies with 1624 subjects were included in the analysis. Patients with BD had statistically significantly larger mean left atrial dimension (0.08, *p* = 0.0008), greater aortic diameter (0.16, *p* = 0.02), significantly reduced ejection fraction (−1.08, *p* < 0.0001), significantly prolonged mitral deceleration time (14.20, *p* < 0.0001), lower *E*/*A* ratio (−0.24, *p* = 0.05), and increased isovolumetric relaxation time (7.29, *p* < 0.00001).* Conclusion.* DD is increased in patients with BD by the presence of several echocardiographic parameters favoring DD as compared to controls. The meta-analysis also identified that LA dimension is increased in BD patients. EF has also been found to be lower in BD patients. Aortic diameter was also increased in BD patients as compared to controls.

## 1. Introduction

Behcet's disease (BD) is a chronic inflammatory disease. BD is seen throughout the world and its estimated incidence ranges from 1 per 100,000 to 70 per 100,000 depending on the geographic region [1, 2]. It can virtually involve any organ system. Cardiac involvement is variable and thought to occur in the range of 7–46% [3] of patients and may cause myocardial infarction, pericarditis, valve problems, aneurysms, or congestive heart failure (CHF).

The development of CHF in BD in itself is rare and secondary to vasculitis which leads to impaired pump function [4]. The fact that patients with BD are relatively young without traditional risk factors and yet can develop these potentially fatal complications merits closer scrutiny and the need to be able to identify the precursor forms of these manifestations.

CHF is a well-known clinical entity which is diagnosed by a combination of clinical criteria and measurements on transthoracic echocardiogram (TTE). Left ventricular diastolic dysfunction (DD) is an important element of CHF as DD may present with its own signs and symptoms or may present as a precursor to overt CHF [5]. The presence of DD in the presence of normal ejection fraction (EF) and presence of symptoms of heart failure is known as diastolic heart failure or as heart failure with preserved ejection fraction [6]. The isolated presence of diastolic mechanical abnormalities on TTE is termed DD and is characterized as one of the stages of heart failure and a target of intervention to prevent progressive heart failure [7].

DD is not a rare disease entity. In the general population, a population based study of more than two thousand research participants reported an overall DD prevalence of 28.1% [8]. Another research paper involving over 36,000 subjects had a DD prevalence of 65.1% with the most common form being that of mild DD (60.0%) [9]. In the general population, DD has been identified as an independent predictor of new onset CHF [10]. It is also a standalone determinant of mortality [8, 9, 11].

DD is a collection of mechanical problems in the contraction and relaxation of the heart. DD has been found to occur in association with diabetes mellitus (DM), increasing age, coronary artery disease (CAD), hypertension (HTN), and overweight/obesity [12]. Increased left atrial (LA) dimension also indicates DD provided there are no concomitant rhythm disorders [11, 13]. Diastolic dysfunction is an echocardiographic diagnosis and not a clinical one.

There is recent literature to support increased cardiac problems in other common rheumatic diseases like rheumatoid arthritis. One recent paper has shown increased DD in rheumatoid arthritis [14]. The data regarding the presence of DD in BD is controversial. Most of these studies were of case-control design and small in size. Our objective was to gather and analyze the available data through a meta-analysis to compare the prevalences of CHF and DD, based on TTE parameters, in patients with BD as compared to healthy controls to try to answer the question of this association more definitively.

## 2. Methods

### 2.1. Literature Search Strategy

The review was conducted according to the recommendations of the meta-analysis of observational studies in epidemiology (MOOSE) group [37]. A time restricted (year 1990 and onwards) and language restricted (English) Medline search with the medical subject heading (MESH) terms “(heart failure OR ventricular dysfunction OR atrial OR echocardiography) AND (behcet OR behcet's disease)" was conducted on 31 December 2013. We also perused the bibliography of the identified studies as well as general review articles on BD to identify any appropriate studies that may have been not selected in the search. Unpublished studies were not included. Bibliographically identified studies were only included if they were also retrievable from the Medline database.

A non-English language search was also performed using the same search strategy but utilizing the language filter properties of Medline. The goal of the non-English language study search was to only review the abstracts and compare the conclusions regarding echocardiographic parameters from the abstracts.

### 2.2. Study Selection

The studies were independently selected by two researchers using our predetermined search strategy. Any differences were resolved by mutual consultation. Studies performed with the intent of evaluating cardiac function between BD patients and controls were included. Studies were only included if the identified patients with BD meet the diagnostic criteria of the international study group for BD [38]. Only case-control studies were included as they provided an odd-ratio estimate of study differences between BD patients and the general population. We included only those studies that had adjusted for or excluded patients with preexisting cardiac conditions. This was to ensure minimal confounding. Studies including patients with overlapping rheumatic disorders were excluded from the study as other rheumatic conditions are known to affect the heart and may confound the results.

Non-English language Medline search revealed 39 studies. Of all these studies only one title merited further scrutiny; however no abstract was available for review.

### 2.3. Study Quality

The Newcastle-Ottawa scale (NOS) for case-control studies was used to assess the quality of the included studies [39]. A higher score is indicative of better methodological quality.

### 2.4. Data Collection

Data was collected pertaining to the sociodemographic, BD related, and laboratory variables. TTE variables included EF in percent (%), isovolumetric relaxation time (IVRT) in millisecond (msec), and mitral valve deceleration time (DT) in msec, transmitral early filling peak velocity (*E*) in meters per second (m/sec), transmitral late filling peak velocity (*A*) in m/sec, and the *E*/*A* ratio. Other variables, where available, included left atrial dimension (LAD) in centimeter (cm), left ventricular end diastolic dimension (LVDD) in cm, left ventricular end systolic dimension (LVSD) in cm, aortic diameter in cm, and aortic distensibility in cm^2^  ×  dyn. Summary estimates were used as individual level patient data was not available.

While the comparison of EF and aortic size was fairly straightforward, that is not the case for DD. A variety of combinations or single values of the aforementioned variables are used to classify patients with DD [40]. The more supportive the variables, the higher the likelihood of having DD. We kept a low threshold and even if one parameter was favoring DD, the subjects were classified as so. It is also to be noted that DD is graded in terms of the degree of impairment into three categories. Such information was not available to us regarding grades of DD.

### 2.5. Statistical Methodology

Since individual patient information was not available for all patients, we report weighted means and standard deviations. Percentages and means ± standard deviations (SD) were calculated to describe the distributions of categorical and continuous variables, respectively. Student's *t*-test for independent samples was used for continuous variables and chi-square test for proportions. The baseline data were analyzed using SAS version 9.3 (SAS Institute, Cary, NC).

Review Manager, version 5.2 (Cochrane Collaboration), was used for the statistical computations of this study. Mean differences (MD) and their 95% confidence intervals (95% CIs) were calculated to determine the effect size for each parameter measured via TTE using the random-effects model. Heterogeneity measurements were calculated and reported by including Cochran's *Q*-statistic, the *I* ^2^ index, and the tau-squared test. Random-effects model was used to calculate the MD and 95% CI. This was done due to the range of heterogeneity in the data.

## 3. Results

A total of 185 citations were identified based on the aforementioned search strategy. Of these, 22 were selected after abstract review (Table 1). A total of 1624 subjects were identified of which 913 (56%) had BD. The mean BD duration was 8.2 years. Pulse wave Doppler (PWD) technique was the most commonly used technique to assess TTE parameters but the newer studies used tissue Doppler imaging (TDI) technique. Mean disease duration was 8.2 years. Mean age of the cases was 37.7 years and that of controls was 37.3 years. Of the cases, 60% were males and 77% of the controls were males. No difference on the basis of smoking, DM, HTN and hyperlipidemia, lipid values, and systolic blood pressure (SBP) was noted. Body mass index (BMI) was statistically significantly higher in the control population (24.8 versus 24.1 kg/m^2^, *p* = 0.037). High density lipoprotein (HDL) was significantly higher in the control population (43.5 versus 41.4 mg/dL, *p* = 0.05) but was likely clinically insignificant based on the small difference. Diastolic blood pressure (DBP) was significantly higher in the cases (76.8 versus 74.8 mmHg, *p* < 0.001). C-reactive-protein (CRP) was significantly higher in the cases (18.1 versus 8.8 mg/dL, *p* < 0.001) as expected.

All studies had participants that were matched on the basis of age and sex and as a general exclusion criterion; subjects with CAD, DM, HTN, and other heart diseases were excluded. Racial data was not available for the studies. The average quality score was 6.4 of a maximum possible of 9 on the NOS for the included studies. Of the 22 studies, 17 showed some difference in TTE parameters between the two groups while 5 showed similar indices. Table 1 gives the details of the studies comprehensively.

Most studies (16 out of 22) reported on medications used in DD patients. Patients on medications known to affect the cardiac functional parameters were usually excluded. As we performed aggregate level data meta-analysis and no individual patient data was reported, we are unable to comment on the effects of medication usage on DD.

**Figure 1:**
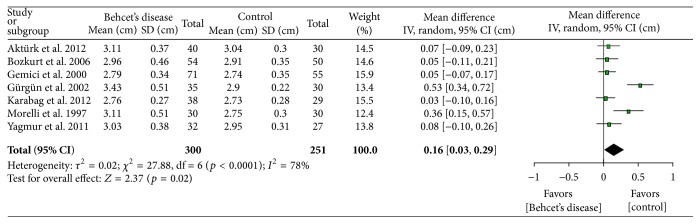
Aortic size comparison between patients with Behcet's disease and controls.

**Figure 2:**
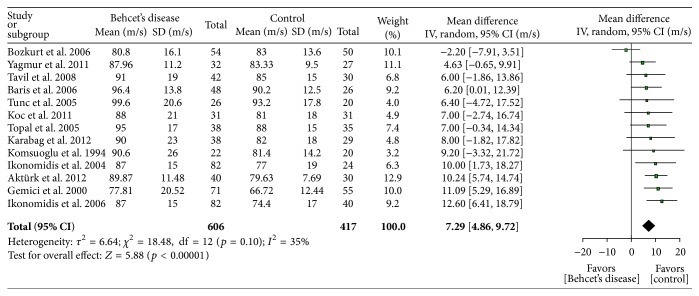
Isovolumetric relaxation time comparison between patients with Behcet's disease and controls.

**Figure 3:**
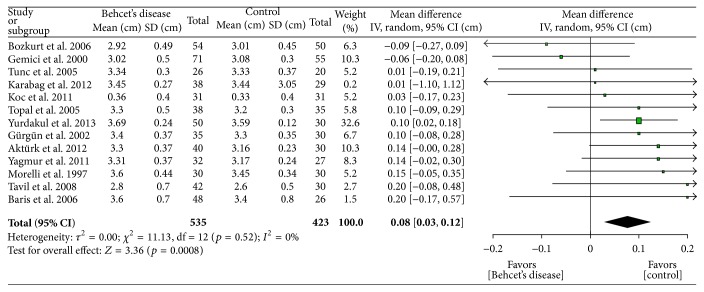
Left atrial dimension comparison between patients with Behcet's disease and controls.

**Figure 4:**
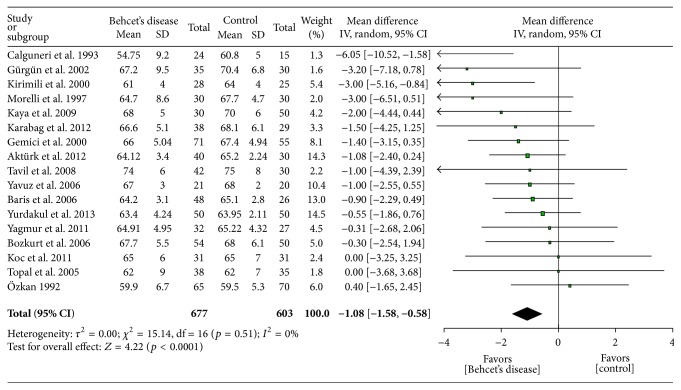
Left ventricular ejection fraction comparison between patients with Behcet's disease and controls.

Table 2 shows the main meta-analysis outcomes. Comparison of the *E*/*A* ratios (figure not shown) shows lower values (−0.24, *p* = 0.05) in BD patients which is an indicator of DD. As expected, aortic diameter (Figure 1) was found to be greater (0.16 cm, *p* = 0.02) in patients with BD than in controls. Larger diameter predisposes to aneurysms. Transmitral *E* velocity (figure not shown) was found to be lower in BD patients (−0.03 cm, *p* = 0.07) suggestive of DD. IVRT (Figure 2) was increased in BD patients (7.29 m/s, *p* < 0.00001) suggesting impaired cardiac relaxation and pointing towards DD. Mitral DT was found to be statistically significantly prolonged (14.20 msec, *p* < 0.0001) in BD patients (figure not shown). LAD was found to be statistically significantly larger (0.08 cm, *p* = 0.0008) in BD patients (Figure 3) suggesting DD. This is also a risk factor for atrial fibrillation [41]. EF of the left ventricle was found to be statistically significantly reduced (−1.08%, *p* < 0.0001) in BD patients (Figure 4) suggesting impaired cardiac function and pumping ability in BD patients as compared to controls.

In terms of other echocardiographic variables, no statistically significant differences were noted between left ventricular mass index, LVSD, LVDD, posterior wall thickness, and left ventricular myocardial perfusion index. With regard to the aortic distensibility, which measures the stiffness of the aortic wall, while the two individual studies [23, 27] reporting this parameter show statistically significant reduced aortic distensibility (figure not shown), the composite outcome was not significant due to the fact that the first study has a much larger effect size and a higher variance than the second study. This heterogeneity leads to a wide confidence interval for the combined results and thus a nonsignificant value.

## 4. Discussion

This systematic review and meta-analysis looking at data from 1624 subjects has clearly shown the presence of DD indices on TTE is greater in patients with BD than with controls. These indices include *E* wave velocity, *E*/*A* ratio, IVRT, and mitral DT. Prolonged mitral DT and IVRT were the most strongly linked DD parameters on TTE in DD patients with BD. Three more important statistically significant findings were that of increased LA size, depressed EF, and increased aortic dimensions in patients with BD as compared to the controls.

The finding of depressed EF reveals the likely presence of a pattern of pathology affecting both systolic and diastolic parameters of the heart. N-terminal brain natriuretic peptide has been found to be elevated in patients with BD signifying the heart strain and elevated risk of cardiac pump problems [42, 43]. This data provides further corroboration of the TTE findings in our study. It must be noted that although the EF was lower in cases as compared to controls, it was not necessarily abnormal.

Increased aortic diameter was seen in this meta-analysis. There are reports of aortitis [44] and inflammation involving the root of the aorta which can lead to subsequent aneurysm formation. Aneurysms can be a major cause of death if they rupture. This finding stresses the importance of following up these patients for further dilation of the aortic root and to take prophylactic measures, if indicated.

One reason for seeing DD in otherwise asymptomatic patients could be a result of the microvascular inflammation that has been seen in cardiac exam and perfusion imaging studies in BD patients [45, 46]. These may affect the hearts mechanical properties through local inflammation and tissue remodeling causing altered TTE indices. Coronary artery vasculitis might also be a contributing factor to this impaired performance through inducing micro level ischemia.

Arterial stiffness is a reliable predictor of cardiovascular mortality and morbidity. It can be assessed by pulse wave velocity (PVW). Central systolic and diastolic pressure can be determined via pulse wave analysis (PWA). An important aspect of wave analysis is wave reflection. Wave reflection depicts the interaction of the outgoing impulse of the cardiac pressure and the reflection from the vascular system. PVW is a marker of arterial stiffness. It has been found to be elevated in patients with BD who have active disease [47]. Nitric oxide (NO) serves as a vasodilator. It is thought that inflammation mediated endothelial dysfunction in BD contributes to increased arterial stiffness. Inflammation mediated release of reactive oxygen species further interferes with NO release and also causes stiffness of cardiac myocytes [6].

It is well known that most of the intrinsic blood flow to the heart occurs during diastole. This is due to the compression of the surrounding musculature. Coronary flow reserve (CFR) is a way to assess the microcirculation of the heart and thereby the endothelial function. This assessment is usually via echocardiography. It is defined as maximal coronary flow divided by basal flow. It can be altered by epicardial pathology and/or microvascular dysfunction. Atherosclerosis is known to have an inflammatory component. Systemic inflammation was thought to play a role in the reduction of CFR in psoriasis patients without any known coronary disease [48]. Reduced CFR has also been found in patients with BD [49], RA [50], and scleroderma [51] thus suggesting the underlying role of inflammation.

The natural history of DD is not very well defined. Studies on the common populace have shown that the factors promoting DD progression to symptomatic stages include ischemia, rhythm problems like atrial fibrillation, hypertension, or hospitalization but remain unknown in 50% of cases [52].

Data from one study of the general population revealed that the two-year likelihood of developing systolic CHF was 1.9% while the likelihood of developing clinical manifestations of heart failure in patients already suffering from DD was at 31.1% [52]. One general population report identified that TTE parameters of DD remained steady in about 50% of the patients, showed improvement in 21%, and based on clinical symptoms deteriorated in 27% after observation for over 3.6 years; and importantly improvement to a less severe degree of DD conferred a survival benefit [11].

There were several studies which assessed the relationship of BD duration to the presence of DD. As is theoretically expected because of accruing damage from the inflammation over the years as well as advancing age promoting coexisting problems, most of these studies reported association of DD and/or aortic diameter with longer BD duration [16, 20, 22, 28, 31, 33, 34]. One study reported on the relationship between the activity state of BD, whether disease was controlled or not, and cardiac manifestations but no significant association was found in that report [24].

The result of increased LA dimension is of great potential importance, because increased LA dimension is a risk factor for atrial fibrillation [41, 53] which in turn confers a high risk of suffering from a stroke. LA dimensions and mechanical problems have also been identified in other autoimmune diseases pointing to a unifying final pathway in these conditions [14, 54]. At present, there is no population level data about the association between atrial fibrillation and BD, but this study has certainly identified an important area for future original research from a clinical health perspective.

Another factor of importance was the concept of electromechanical delay (EMD) identified in one of the studies of this meta-analysis [17]. This concept basically implies that it takes a longer time for the electrical energy generated in the heart muscle to transform into mechanical energy which then leads to the contraction of the heart. Increased EMD is a well-established predictor of development of atrial fibrillation [55]. Studies have previously also shown findings of prolonged atrial signal travelling time in BD cases which was related to the length of their disease process [56]. The finding of increased EMD in BD also further corroborates the potential for increased risk of atrial arrhythmia [17]. It is believed that the vasculitis process in BD leads to fibrosis of the cardiac and atrial muscles which interferes with the passage of electrical signal in the atrial musculature leading to aberrant circuit formation [17]. Measuring EMD by TDI may be a simple and useful way to analyze the risk of atrial fibrillation in patients with BD.

### 4.1. Treatment Implications and Suggestions for Future Research

At present there is no targeted and specific treatment for DD. The focus is on the control of the risk factors that may lead to DD and its progression to overt CHF. Studies are underway to determine appropriate treatment strategies for these patients [5]. Aggressive blood pressure control [57] may improve TTE diastolic indices [58]. It is important for physicians managing patients with BD to consider their higher risk of developing DD and CHF and be more aggressive in the control of traditional risk factors if present. However, since BD is relatively a disease of the young and these patients may not have developed the traditional risk factors thus their cardiac health may not be assessed. At present, it is not clear whether these risk factors should be more actively sought in these patients or not. Also it is not clear as to how to follow up these patients, especially if asymptomatic, with echocardiograms, and if they do have subtle findings, how to go about addressing them. These are important areas for future research and designing of health services. Further future studies could look at the activity of the disease and relate it to development of cardiac problems. Additional studies could be designed to compare BD patients who develop cardiac complications with those who do not and try to ascertain the determinants of the differences.

### 4.2. Strengths of the Study

Our analysis included mostly subjects without any of the known medical conditions that are risk factors for DD. All studies were of decent quality according to the NOS scale quality and had the primary objective planned to evaluate TTE parameters in BD. It is important to note that most studies had excluded patients with known cardiotoxic medications which could have confounded the results. One strength of our data is that it has identified many TTE parameters, rather than just one, pointing towards DD in these patients and this lends strength to the concept that DD is increased in BD patients. We also searched for non-English language studies and were not able to identify any relevant studies based on title and abstract review. We hope this search will serve to limit the non-English language publication bias.

### 4.3. Limitations of the Study

Our study has some limitations. Unknown factors can always be a source of bias and may cause confounding. Observer bias may have contributed as most of the papers did not mention if the TTE operator was blinded to the case-control status of the patients. Publication bias against negative studies could not be excluded.

In clinical practice, DD diagnosis is based on several TTE values and not one solitary abnormality and the reliance on only one abnormal value may have elevated the degree of presence of DD in our paper. Also, our values do not meet any predefined cut-off values favoring DD but just that the TTE indices are more inclined towards DD in BD patients and these are statistically significant. We were unable to report on the grade of DD as it was mostly not reported or not possible to combine in the analysis. As we discussed earlier, the grade of DD is important from a prognostic perspective.

We did note that our cases had slightly higher DBP values than controls. Controls had higher HDL levels and BMI. While BMI was higher in the control group, its average was within the normal range and likely was not a contributing factor to the results. And even if it were, it would likely have only served to reduce the difference between the two groups by increased values of the TTE measurements in the control population. HDL level at present seems to have no robust influence on DD. It may be argued that the higher DBP might have confounded the results but we do not believe so due to the fact that average DBP was within the normal range and that SBP is the more important determinant from the pathology and management perspective, and it was similar in the two groups [59].

As there was a considerable amount of heterogeneity in the papers used in the meta-analysis, we utilized random-effects models to limit the effects of heterogeneity on the results of our meta-analysis. This heterogeneity was a result of different patient populations, clinical environment, TTE performers, BD severity, and procedure of control selection. The results of this aggregate data meta-analysis may not be applicable to the singular patient as no individual level data was ascertainable. Most of the data is confined to studies from Turkey which is the country that has the most experience with BD and thus may not be applicable universally. We must, however, consider that BD is a disease with geographic risk factors and is expected to be seen more in certain areas than the others. We were also not able to analyze the association between the degree of BD activity or severity and the cardiac manifestations.

## 5. Conclusions

This meta-analysis has combined data from the all the published studies on the topic and identified that DD is increased in patients with BD by the presence of several TTE parameters favoring DD as compared to controls. It has also identified that LA dimension is increased in BD patients. EF has also been found to be lower in BD patients. Future longitudinal studies could follow the natural history of cardiac function in BD patients and see the rate of natural progression of DD to overt CHF and compare it to the trend in the general population.

## Figures and Tables

**Table 1:** General study characteristics and major findings.

Number	Author [reference] (country)	Subjects	Age/sex match	ISG criteria	Major results	Notes	BD year (mean)	CAD	Active medications	TTE blind	NOS^*∗*^
1	Yurdakul et al. [15] (Turkey) 2013	Total: 80 BD: 50	Y/Y	Y	Subclinical left and right ventricular systolic dysfunction in BD patients	—	5.9	N	NA	Y	2/2/3

2	Aktürk et al. [16] (Turkey) 2012	Total: 70 BD: 40	Y/Y	Y	Increased LA volume and DD in BD patients	Disease duration and CRP levels were predictive	4.9	N	NA	Y	2/2/3

3	Karabag et al. [17] (Turkey) 2012	Total: 67 BD: 38	Y/Y	Y	More DD and prolonged atrial conduction times in BD patients	—	10.5	N	Colchicine, prednisolone & cyclosporine	NA	3/2/3

4	Cobankara et al. [18] (Turkey) 2012	Total: 48 BD: 24	Y/Y	Y	Normal LV MPI and increased RV MPI in BD patients	—	NA	N	NA	Y	4/2/3

5	Yagmur et al. [19] (Turkey) 2011	Total: 59 BD: 32	Y/Y	Y	Significant impairment of mean longitudinal strain and higher DT in BD patients	Elevated NT-proBNP in BD patients	5.37	N	Cardiotoxic medicines excluded. Colchicine	NA	2/2/2

6	Koc et al. [20] (Turkey) 2011	Total: 62 BD: 31	Y/Y	Y	More DD and larger LA in patients with BD	Increased PWD in BD patients. Disease duration correlated with DD and PWD	5.0	N	Cardiotoxic medicines excluded	Y	1/2/3

7	Kaya et al. [21] (Turkey) 2009	Total: 80 BD: 30	Y/Y	Y	Similar EF in both groups	Lower heart rate recovery index in BD patients	11.0	N	Cardiotoxic medicines excluded	NA	2/2/2

8	Tavil et al. [22] (Turkey) 2008	Total: 72 BD: 42	Y/Y	Y	LV MPI decreased in BD patients	No influence of colchicine	2.7	N	Colchicine, prednisolone, azathioprine, salicylates & cyclosporine	Y	2/2/3

9	Ikonomidis et al. [23] (Greece) 2006	Total: 122 BD: 82	Y/Y	Y	Prolonged IVRT in BD patients	BD patients had impaired aortic distensibility and central augmentation index	10.0	N	Steroids and antihypertensive	NA	2/2/2

10	Yavuz et al. [24] (Turkey) 2006	Total: 41 BD: 21	Y/Y	Y	LV and RV DD more in BD patients	No association with disease activity or medicine usage	10.0	N	Colchicine, prednisolone, azathioprine & cyclosporine	NA	2/2/2

11	Baris et al. [25] (Turkey) 2006	Total: 73 BD: 48	Y/Y	Y	More DD in BD patients	DD based on *E*/*A* ratio only	8.09	N	Cardiotoxic medicines excluded	NA	1/2/2

12	Bozkurt et al. [26] (Turkey) 2006	Total: 104 BD: 54	Y/Y	Y	No difference in systolic or diastolic function between the two groups	No difference by TTE technique	8.3	N	Cardiotoxic medicines excluded. Colchicine	NA	2/2/2

13	Tunc et al. [27] (Turkey) 2005	Total: 46 BD: 26	Y/Y	Y	No difference in diastolic function between the two groups except for increased mitral DT in BD	Increased aortic stiffness in BD patients	7.1	N	Cardiotoxic medicines excluded. Colchicine	NA	2/2/2

14	Topal et al. [28] (Turkey) 2005	Total: 73 BD: 38	Y/Y	Y	More DD in BD patients	Diastolic parameters correlated with disease duration	NA	N	Cardiotoxic medicines excluded	Y	2/2/3

15	Ikonomidis et al. [29] (Greece) 2004	Total: 106 BD: 82	Y/Y	Y	More DD in BD patients	Increased aortic stiffness and diameters in BD patients. Prolonged DT is a marker of vascular complications	10.0	N	Cardiotoxic medicines excluded. Colchicine, prednisolone, azathioprine, cyclosporine & statins	NA	2/2/3

16	Gürgün et al. [30] (Turkey) 2002	Total: 65 BD: 35	Y/Y	Y	No data on DD given for comparison. General indices similar between two groups	Significant valvular pathology in BD patients. Increased QT dispersion and larger aortic diameter also noted	8.0	N	NA	Y	2/0/3

17	Gemici et al. [31] (Turkey) 2000	Total: 126 BD: 71	Y/Y	N	Increased DD in BD patients	Increased repolarization dispersion in BD patients. This may be related to DD. DD also related to disease duration	9.6^*∗*^	NA	NA	NA	2/0/2

18	Kirimli et al. [32] (Turkey) 2000	Total: 53 BD: 28	Y/Y	Y	Increased DD in BD patients	Increased repolarization dispersion in BD patients	11.0	N	Cardiotoxic medicines excluded. Colchicine, prednisolone & cyclosporine	Y	3/2/3

19	Morelli et al. [33] (Italy) 1997	Total: 60 BD: 30	Y/Y	Y	Increased aortic diameter in BD patients. Normal ventricular function in both groups	Significant valvular pathology in BD patients. Diameter associated with disease duration. Did not look for DD	10.0	N	NA	Y	3/0/3

20	Komsuoglu et al. [34] (Turkey) 1994	Total: 42 BD: 22	Y/Y	Y	Increased DD in BD patients	DD related to disease duration	5.0	N	Cardiotoxic medicines excluded. Colchicine, steroids, antiaggregant & other anti-inflammatories	NA	2/1/2

21	Calguneri et al. [35] (Turkey) 1993	Total: 39 BD: 24	Y/NA	N	No major difference in TTE but seen on radionuclide imaging	—	NA	N	Colchicine, steroids, antiaggregant & other anti-inflammatories	NA	2/1/2

22	Özkan et al. [36] (Turkey) 1992	Total: 135 BD: 65	Y/Y	Y	No difference in TTE parameters	DD not specifically studied	5.7	N		Y	4/1/3

^*∗*^The first section reflects score out of 4 for subject selection, the second section after the forward slash reflects subject comparability score out of two, and the final section after the second forward slash reflects score out of 3 for exposure ascertainment.

**Table 2:** Meta-analysis outcomes (random-effects model).

Variables	Mean difference (95% CI)	*p*	*Q* ^*∗∗*^	*I* ^2^, %^*ǂ*^	τ^2^^§^
Left atrial dimension, cm (*n* = 958)	0.08 (0.03, 0.12)	0.0008	11.13	0	0.00
Left ventricular mass index, gm/m^2^ (*n* = 300)	3.53 (−1.08, 8.14)	0.13	0.11	0	0.00
Isovolumetric relaxation time, msec (*n* = 1023)	7.29 (4.86, 9.72)	<0.0001	18.48	35	6.64
Transmitral *A* wave velocity, meters/second (*n* = 705)	0.03 (−0.01, 0.08)	0.18	37.21	79	0.00
Transmitral *E* wave velocity, meters/second (*n* = 705)	−0.03 (−0.06, 0.00)	0.07	15.04	47	0.00
*E*/*A* ratio (*n* = 304)	−0.24 (−0.48, 0.00)	0.05	36.50	89	0.06
Mitral deceleration time, msec (*n* = 1023)	14.20 (7.67, 20.73)	<0.0001	32.96	34	82.96
Left ventricular ejection fraction, % (*n* = 1280)	−1.08 (−1.58, −0.58)	<0.0001	15.14	0	0.00
Posterior wall, cm (*n* = 682)	0.00 (−0.03, 0.03)	0.81	25.01	64	0.00
Left ventricular end systolic dimension, cm (*n* = 742)	0.03 (−0.04, 0.10)	0.37	18.03	45	0.01
Left ventricular end diastolic dimension, cm (*n* = 874)	−0.02 (−0.08, 0.04)	0.46	16.31	26	0.00
BSA, m^2^ (*n* = 80)		0.12			
LV MPI (*n* = 200)	0.04 (−0.07, 0.16)	0.48	47.96	96	0.01
Aorta, cm (*n* = 551)	0.16 (0.03, 0.29)	0.02	27.88	78	0.02
Aortic distensibility (*n* = 168)	−1.23 (−3.06, 0.60)	0.19	27.69	96	1.69
Systolic blood pressure, mmHg (*n* = 994)	1.69 (0.16, 3.22)	0.03	14.59	11	0.92
Diastolic blood pressure, mmHg (*n* = 941)	1.34 (0.29, 2.39)	0.01	12.69	5	0.20
Heart rate (*n* = 927)	0.68 (−0.76, 2.13)	0.35	29.14	59	2.85
Males, % (*n* = 1199)	0.96 (0.87, 1.06)^*∗*^	0.39	4.16	0	0.00
Age, mean years (*n* = 1238)	−0.40 (−1.32, 0.53)	0.40	17.98	5	0.22
BMI, kg/m^2^ (*n* = 831)	−0.27 (−0.82, 0.27)	0.32	16.32	39	0.29
Diabetes mellitus (*n* = 295)	1.31 (0.42, 4.05)^*∗*^	0.64	0.17	0	0.00
Hypertension (*n* = 373)	0.92 (0.52, 1.65)^*∗*^	0.79	0.30	0	0.00
Hyperlipidemia (*n* = 154)	1.15 (0.50, 2.64)^*∗*^	0.74	0.62	0	0.00
Smoking (*n* = 728)	0.97 (0.83, 1.15)^*∗*^	0.76	5.04	0	0.00
Low density lipoprotein, mg/dL (*n* = 465)	−0.72 (−7.48, 6.05)	0.84	22.89	74	54.06
HDL, mg/dL (*n* = 433)	−0.91 (−3.38, 1.56)	0.47	13.30	55	5.44
Triglycerides, mg/dL (*n* = 550)	−1.35 (−10.86, 8.16)	0.78	11.79	49	72.83
Total cholesterol, mg/dL (*n* = 637)	−0.65 (−7.16, 5.85)	0.84	13.91	42	36.40
HS-CRP, mg/dL (*n* = 268)	7.79 (1.89, 13.68)	0.01	10.47	71	24.27

^*∗*^Risk ratio.

^*∗∗*^Cochran's *Q*-statistic for heterogeneity.

^**ǂ**^ *I* ^2^ index for degree of heterogeneity.

^§^Tau-squared measure of heterogeneity.
